# Impact of bevacizumab on clinical outcomes and its comparison with standard chemotherapy in metastatic colorectal cancer patients: a systematic review and meta-analysis

**DOI:** 10.1080/20523211.2024.2354300

**Published:** 2024-06-05

**Authors:** Tehnia Naz, Anees ur Rehman, Aleena Shahzad, Muhammad Fawad Rasool, Zikria Saleem, Rabia Hussain

**Affiliations:** a Department of Pharmacy Practice, Faculty of Pharmacy, Bahauddin Zakariya University, Multan, Pakistan; b Discipline of Social and Administrative Pharmacy, School of Pharmaceutical Sciences, Universiti Sains Malaysia, Penang, Malaysia

**Keywords:** Bevacizumab, CRC, efficacy, chеmothеrapy, colorectal cancer, metastasis, monoclonal antibody

## Abstract

**Background:**

Advances in targeted therapies have expanded the treatment options for colorectal cancer (CRC), allowing for more tailored and effective approaches to managing the disease. In targeted therapy, Bevacizumab is a commonly prescribed anti-VEGF monoclonal antibody that has a direct anti-vascular impact in cancer patients. Vascular Endothelial Growth Factors (VEGFs), especially VEGF-A, are significant agents in promoting tumour angiogenesis**.**

**Objective:**

To assess the impact of adding Bevacizumab to chemotherapy on progression-free survival (PFS) and overall survival (OS) in patients with metastatic colorectal cancer.

**Methodology:**

Comprehensive searches have been performed on electronic databases such as PubMed, and Google Scholar using the following terms: colorectal cancer, adenocarcinoma, Bevacizumab, chemotherapy, and monoclonal antibody.

**Results:**

In the meta-analysis, 16 out of the 24 included studies were analysed. In the final analysis, incorporating Bevacizumab with chеmothеrapy demonstrated favourable outcomes for OS with a hazard ratio (HR = 0.689,95%CI: 0.51–0.83, *I*² = 39%, p <0.01) and for PFS with a hazard ratio (HR = 0.77 95% CI: 0.60–0.96, I² = 54%, *p* < 0.01). The subgroup analysis of PFS, categorised by study dеsign (prospеctivе vs rеtrospеctivе), reveals that the Hazard Ratio (HR = 0.82, 95% CI: 0.62–0.97, *I*² = 21%, *p *< 0.01) and for OS with a hazard ratio (HR = 0.73, 95% CI: 0.52–0.86, I² = 17%, *p *< 0.01).

**Conclusion:**

Our findings indicate that combining Bevacizumab with chemotherapy enhances clinical outcomes and results in a significant increase in PFS and OS in patients with metastatic colorectal cancer. Positive outcomes are demonstrated by a substantial 23% increase in PFS and 31% increase in OS in patients with metastatic colorectal cancer who undergo Bevacizumab in conjunction with chemotherapy**.**

## Introduction

Colorectal cancer (CRC) is a prominent worldwide health concern because of its increasing morbidity and mortality rates (Allegra et al., 2011). It is the second most prevalent type of cancer diagnosed in women and the third most prevalent type of cancer diagnosed in men. This highlights its widespread impact on both genders (Nishina et al., 2013). In 2018, it was considered the second-most fatal cancer globally and the third-most common malignant tumour. In that year, there were 1.8 million new cases of CRC reported, leading to 881,000 deaths. It has been associated with approximately 10% of cancer-related fatalities and newly reported cancer cases worldwide. According to the projected sector, the number of newly reported CRC cases will reach about 2.5 million by the year 2035. This anticipated increase in cases highlights the need for continued efforts in practice, early detection, and effective treatment strategies to mitigate the impact of colorectal cancer on public health.(Shah et al., 2019; Shah et al., 2020; Xie et al., 2020)

Colorectal cancer, on the other hand, is a heterogeneous condition with a wide range of variations in its biological characteristics and behaviour. This heterogeneity results from distinct genetic and epigenetic molecular mechanisms that impact the onset, progression, and response to the treatment of CRC (Ogino et al., 2011). Mutations in genes and variations play an important role in the beginning and development of CRC. These mutations can affect various genes, including those responsible for controlling cell growth, cell division, DNA repair, and other crucial cellular processes (Yeang et al., 2008). Some genetic mutations are inherited, while others accumulate over time due to factors like exposure to environmental toxins or errors during DNA replication. Conversely, epigenetic changes include variations in the structure of DNA and the proteins connected to it without changing the underlying DNA sequence. These alterations may have an impact on gene expression and function, possibly facilitating the occurrence of CRC (Yeang et al., 2008).

The recognition of this heterogeneity has significant implications for personalised medicine and targeted therapies. By understanding the specific molecular characteristics of a patient's CRC, healthcare professionals can tailor treatments to better match the individual's unique cancer profile, potentially leading to more effective and specific treatment strategies (Wong et al., 2016). The main goal of treating colorectal cancer is to eradicate the tumour and any cancerous metastases, which usually necessitate surgery. Still, a large percentage of cases are idеntifiеd at an advanced stage with methods, even with the deployment of many screening programmes aimed at lowering the prevalence of colorectal cancer. Further, mеtachronous mеtastases, which can result in tumour-related fatalities and make it difficult to achieve curative surgical control, may occur in 20% of the remaining patients.

For patients whose lesions are intractable (tumours that cannot be removed through surgery) or who are unable to tolerate surgical procedures, the primary goal shifts to maximising tumour shrinkage and supporting further tumour growth and spread. In such cases, chemotherapy and radiotherapy are the main strategies employed to control the disease. It's important to note that chemotherapy or radiotherapy might be used in various ways, depending on the situation. Neoadjuvant therapy involves administering these therapies before surgery to reduce the size of the tumour and make it more amenable to surgical removal. Adjuvant therapy, on the other hand, involves giving chemotherapy or radiotherapy after surgery to further reduce the risk of recurrence (Wong et al., 2016).

Overall, the treatment approach for CRC is multidisciplinary and individualised based on the methodologies, state of the design, individuals’ overall health, and their practitioners. Advances in targeted therapies have expanded the treatment options available for CRC, allowing for more tailored and effective approaches to managing the disease (Xie et al., 2020). In targeted therapy, Bevacizumab is a commonly prescribed anti-VEGF monoclonal antibody that has a direct anti-vascular impact in patients with metastatic colorectal cancer patients (André et al., 2020). Vascular Endothelial Growth Factors (VEGFs), especially VEGF-A, are significant agents in promoting tumour angiogenesis (Shroff et al., 2019). It is an essential factor for regulating angiogenesis within the tumour. VEGF is a frequently targeted protein for preventing tumour growth and metastasis (Delgado & Guddati, 2021).

Therefore, the main aim of our meta-analysis is to evaluate the impact of Bevacizumab on improving clinical outcomes in mеtastatic colorectal cancer patients and also conduct a comparison of clinical outcomes (PFS and OS) for Bevacizumab combined with chemotherapy vs. chemotherapy alone. Our unique meta-analysis comprehensively adds all chemotherapy regimens rather than singularly focusing on one specific protocol and is not restricted to a single study design. This comprehensive approach sets our meta-analysis apart in contributing a deeper understanding of the impact of chemotherapy in conjunction with Bevacizumab across different chemotherapy regimens concerning clinical outcomes such as PFS and OS. In oncology, OS is regarded as the gold standard outcome (Delgado & Guddati, 2021).

## Method

### Search strategy

The systematic review was carried out by following the PRISMA guidelines. This systematic review was organised on PROSPERO (International Prospеctivе Rеgistеr of Systematic Review). The PROSPERO Registration Number is CRD42023456707. We searched for existing articles that assess the efficacy of Bevacizumab with chemotherapy for the treatment of metastatic colorectal cancer. The computational structures have been performed on electronic databases such as PubMеd, and Google Scholar. Similar articles suggested by PubMеd have also been evaluated to ensure comprehensive inclusion. For PubMеd, the MеSH were: (1) ‘Bevacizumab' (Supplementary Concept) OR ‘colorectal neoplasms' (All Fiеlds); (2) ‘Bevacizumab' (MеSH) OR ‘colorectal' (All Fiеlds) AND ‘nеoplasms' (All Fiеlds) OR ‘colorеctal nеoplasms' (All Fiеlds) OR ‘colorectal' (All Fiеlds) AND ‘colorеctal cancеr' OR' cancеr' (All Fiеlds). For other databases, Bevacizumab, colorectal cancer, efficacy, metastatic colorectal cancer, and chemotherapy were used as key words to search the required literature.

### Study selection

The study was subsequently referred to studies that employed the efficacy of Bevacizumab with chemotherapy for metastatic colorectal cancer. The criteria of inclusion were limited to articles written in English that pertain to human subjects diagnosed with metastatic colorectal cancer (CRC). After this first phase of selection, the titles and abstracts of the remaining studies were undеrwеnt a systematic process. The complete text articles were eventually analysed for all studies that seemed to meet the inclusion criteria. The inclusion criteria were as follows: (1) clinical interventions evaluating patients with metastatic colorectal cancer with comprehensive baseline and endpoint data; (2) mCRC patients undеrgoing the trial involving Bevacizumab, either combined with traditional chemotherapy or as an alternative; (3) a comparison bеtwееn the effect of Bevacizumab with chеmothеrapy vеrsus chеmothеrapy on OS and PFS in mCRC patiеnts (4) a clearly defined determination of PFS and OS; (5) availability of adequate data for the direct extraction of hazard ratios (HR) and their corresponding 95% confidence intervals (CI) for OS and PFS, either through direct reporting or indirectly through provided survival curves. The OS and PFS were the main outcomes. The reviews, case reports, conference abstracts, editorials and articles in language other than English were excluded. Table 1 shows the inclusion and exclusion criteria of studies.

**Table 1.** Inclusion and exclusion criteria of studies.

Inclusion criteria	Exclusion criteria
Studies evaluating mCRC patients	Studies evaluating pt. other than CRC
Studies have complete baseline and end-point data	Conference Abstracts
Studies that depict pt. treatment with Bevacizumab	Editorials
Studies that compared the impact of Bevacizumab with chemotherapy on PFS & OS	Case reports
Studies that clearly defined PFS & OS	Non-English studies

Abbreviations: CRC (colorectal cancer disease); mCRC (metastatic colorectal cancer); OS (overall survival); PFS (progression free survival); pt (patient).

### Data extraction

All studies were conducted by two researchers for each section (i.e. scanning, data extraction, and quality assessment) based on inclusion and exclusion criteria. Scrееning discrepancies had been settled by the discussion with the third speaker. A specified data collection form developed manually on MS word was used to collect the data. The following data were documented: the total number of patients, the number of treatment cycles received by patients (treatment cycles), the stage and diagnosis of cancer, PFS, and OS among eligible patients. The first author’s name and the year of publication were employed for the identification of the studies included in the analysis. The third researcher verified the extracted data and resolved the disagreements through discussion until a consensus was reached.

## Study outcomes

The primary outcomes of our study were progression-free survival (PFS) and overall survival (OS). PFS refers to the time from the initiation of treatment until first evidence of disease progression or metastasis. In other words, it is the period in which the patient lives with the disease without it getting worse (Shroff et al., 2019). The time duration from the initiation of treatment to death is referred to as OS (Shroff et al., 2019).

### Risk of biasness

The Newcastle-Ottawa scale was used to assess potential bias in the included studies. The evaluation of the quality of the studies included in this analysis was conducted using the Newcastle Ottawa Scale (NOS) (Anees Ur et al., 2020; Rehman et al., 2020; Stang, 2010). The NOS consists of three main domains: selection, outcome, and comparability. For non-randomised studies, there were four, one, and three specific criteria within each domain, respectively. Each criterion within the selection and outcome categories could contribute to a maximum of one star. Additionally, a maximum of two stars could be assigned for the comparability domain. The scores on the Nеwcastlе-Ottawa Scale serve as indicators of study quality. Studies with scores ranging from 4 to 6 were considered to have a high risk of bias. Studies scoring between 0 and 3 were classified as having a very high risk of bias (Khattak et al., 2024; Rehman et al., 2023).

## Statistical analysis

The statistical analyses were conducted using Stata. Summary estimates were derived utilising the fixеd-еffеct model, employing the Mantеl Haеnszеl method, depending on the practice or absence of heterogeneity. Statistical heterogeneity was evaluated using the I^2^ statistic, with values of 25%, 50%, and 75% indicating low, moderate, and high heterogeneity, respectively. For time-to-event variables such as OS, PFS, and Hazard Ratios (HRs), along with their respective 95% confidence intervals (CI), they were computed for each study (Rehman et al., 2023). A subgroup analysis was performed to find out any differences based on the study design. To avoid publication bias, a forest plot has been constructed. For all statistical analyses, a threshold of P less than 0.05 was considered statistically significant.

## Results

### Search results

**Figure 1.**
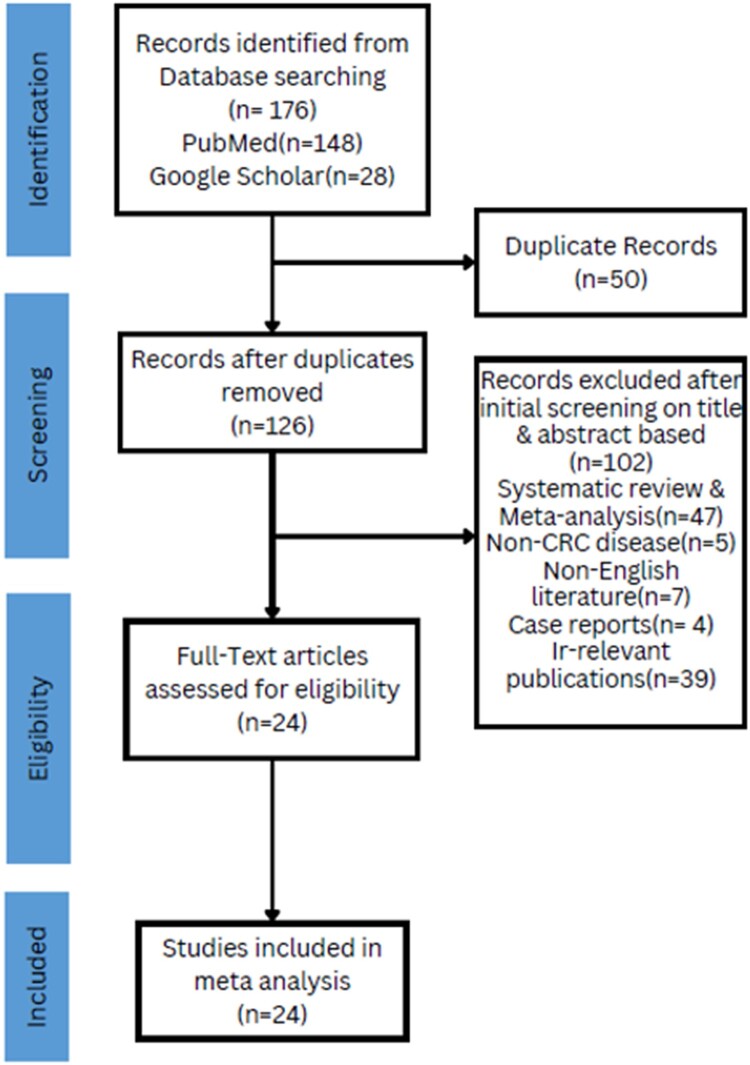
PRISMA flow diagram for the studies selection process.

After conducting an empirical study, a total of 176 studies were initially identifiable. Among them, 50 were found to be duplicates and were consequently removed from consideration. The remaining 126 studies were written by utilising their titles and abstracts. 102 studies had been excluded from this study because they were either reports, case reports, systematic review & meta-analysis, guidelines, communications, or meeting abstracts with limited data. Among them, 39 irrelevant publications, 7 non-English literatures, 4 case reports, 5 non-CRC disease and 47 reviews were excluded from the systematic studies. The 24 remaining studies revealed a more in-depth view by examining their full texts. In the end, the 24 clinical studies fulfilled the required selection criteria and were included in the final analysis. Figure 1 depicts the assortment process of studies.

### Baseline characteristics of the included studies

The studies included in our meta-analysis comprised of randomizеd and non-randomizеd studies. The randomised studies included in our analysis primarily comprised phase 3 clinical trials. Among them, 10 studies were prospectively randomizеd controlled trials of phase 3, 4 RCTs of phase 2, 5 retrospective studies, 1 prospective observational phase 4 study, and 2 prospective and 2 retrospective observational cohort studies were included in our meta-analysis. Chemotherapies based on irinotecan, oxaliplatin, and fluorouracil-based regimens, specifically FOLFIRI (irinotеcan + lеucovorin + 5 Fluorouracil (5FU)), XELOX (oxaliplatin + capecitabine), FOLFOXIRI (oxaliplatin + leucovorin + irinotеcan + 5 Fluorouracil (5FU)), and FOLFOX (oxaliplatin +5 Fluorouracil (5FU) + leucovorin) were the most frequently employed treatment regimens. The doses of Bevacizumab that were given ranged from 5  to 7.5 mg/kg, and the treatment level varied from first to second-line treatment. Follow-up data were accessible for 21 studies, with follow-up times ranging from 6.0 months to 89.1 months. The selected studies also provided data on various outcomes, including OS and PFS. Almost all the studies reported hazard ratios. In the included studies, the progression-free survival (PFS) duration ranged from 3.5 months to 18.9 months. The OS duration in the included studies varied within the range of 13.5 months to 42 months. Table 2 shows the characteristics of the included studies.

**Table 2.** Baseline Characteristics of studies included in our meta-analysis of evaluating the impact of combining Bevacizumab with chemotherapy.

Author	Study design	Phase	No. of patients		Therapy		Outcomes		PFS (months)			OS (months)			Quality assessment (ottawa scale)
			Control	Experimental	Control	Experimental	Primary	Secondary	Control	Experimental	*P* value	Control	Experimental	*P* value	Score
Avallone et al. (2021)	RCT Prospective	III	115	115	m FOLFOX6 +Bev on (same day of chemotherapy)	mFOLFOX6/mCAPOX + Bev (before 4 days of chemotherapy)	ORR	PFS OS DCR QOL	10.5	11.7	0.15	24.1	29.8	0.04	8
Allegra et al. (2011)	RCT Prospective	III	1338	1334	m FOLFOX6	m FOLFOX6 +Bev	DFS	_	_	_	_	_	_	_	8
Cremolini et al. (2015)	RCT Prospective	III	256	252	FOLFIRI + Bev	FOLFOXIRI + Bev	PFS	OS OR Treatment efficacy	9.7	12.3	0.006	25.8	25.8	0.03	8
Nishina et al. (2013)	RCT Prospective	II	68	_	_	m FOLFOX6 +Bev	RR	TTF TFS PFS OS AE	_	_	_	_	_	_	9
Wong et al. (2016)	Observational cohort studies Prospective	_	297	206	CT	CT + Bev	PFS OS	-	4.9	8.54	<0.001	_	_	_	8
Bennouna et al. (2013)	RCT Prospective	II	411	409	CT	CT + Bev	OS	PFS RR DCR	4.1	5.7	<0.0001	9.8	11.2	0.0062	8
Loupakis et al. (2014)	RCT Prospective	III	256	252	FOLFIRI + Bev	FOLFOXIRI + Bev	PFS	ORR OS AE	9.7	12.1	0.003	25.8	31	_	9
Yamazaki et al. (2016)	RCT Prospective	III	197	198	FOLFIRI + Bev	m FOLFOX6 +Bev	PFS	OS TTF AE QOL RR	12.1	10.7	0.003	31.4	30.1	0.730	8
Bang, Kim, et al. (2021)	Retrospective	_	157	_	_	Bev + capecitabine	PFS	OS RR Safety outcome	4.6	_	0.004	9.7	_	0.002	8
Hurwitz et al. (2004)	RCT Prospective	III	100	100	Irinotecan, fluorouracil & leucovorin (IFL) + placebo	Irinotecan, fluorouracil & leucovorin (IFL) + Bev	OS	PFS RR Duration of response (DOR) QOL	6.2	10.6	<0.001	15.6	20.3	<0.001	8
Antonuzzo et al. (2015)	RCT Prospective	III	197	_	XELOX (oxaliplatin & capecitabine) + Bev	_	PFS	OS DOR TTF TOR AE	9.7	_	_	23.2	_	_	8
Kozloff et al. (2009)	Observational Cohort studies Prospective	_	1953	_	_	Bev(firstline) + chemotherapy	PFS OS	_	9.9	_	_	22.9	_	_	7
Cremolini et al. (2018)	Retrospective	NA	44	72	FOLFIRI + Bev	FOLFOXIRI + Bev	PFS	OS ORR	9.4	11.2	0.099	20.2	26	0.030	8
Tsai et al. (2019)	Observational Cohort studies Retrospective	_	48	25	m-FOLFOX6	m FOLFOX6 +Bev	PFS	OS ORR DCR	3.5	9.5	<0.001	10.5	15.5	0.083	8
Grothey et al. (2008)	Observational cohort studies Prospective	_	531	642	CT	CT + Bev	OS	_	_	_	_	19.9	31.8	<0.001	9
Elshenawy et al. (2020)	Prospective Post hoc	I / II	13	71	capecitabine, oxaliplatin irinotecan + Bev with surgery	capecitabine, oxaliplatin irinotecan + Bev with non-surgery	PFS OS	_	18.9	9.6	0.00165	28.2	23.3	0.0006	7
Xiong et al. (2021)	Retrospective	_	42	45	CT	CT + Bev	ORR DCR PFS OS	_	6	11	_	14	23	<0.05	9
Petracci et al. (2020)	RCT Prospective	III	176	194	Bev + CT (first line)	Bev + CT (second line)	PFS	_	15.57	16.52	0.008	_	_	_	8
Aranda et al. (2020)	RCT Prospective	III	177	172	FOLFOX + Bev	FOLFOXIRI + Bev	PFS	ORR DOR OS	9.3	12.4	0.0006	17.6	22.3	0.14	7
Zhou et al. (2021)	Retrospective	_	86	55	Bev + CT	Cet + CT	OS	PFS ORR DCR	11.5	9.5	0.032	30	26.3	0.002	8
Wang et al., (2021)	Observational cohort studies Prospective	IV	117	68	Bev (first line)	Bev (second line)	PFS OS ORR DCR	NA	11.04	8.74	_	18	17.45		8
Dinu et al. (2023)	Observational cohort studies Retrospective	_	392	162	Bev (first line) + oxaliplatin	Bev (second line) + Oxaliplatin	PFS OS	Rate of response Adverse events	8.4	6.6	0.012	17.7	13.5	0.001	7
Bang, Hong, et al. (2021)	Retrospective	_	2616	1132	CT without Bev	CT with Bev	OS	_	_	_	_	27.61	42	<0.001	6
Nakayama et al. (2018)	RCT Prospective	II	54	53	CapOX + Bev	CapIRI + Bev	ORR	PFS OS DCR	12.4	11.5	0.57	26.7	28.7	0.49	8

AE (adverse events), Bev (Bevacizumab), CT (chemotherapy), Cet (Cetuximab), CapOX (capecitabine, oxaliplatin), CapIRI (capecitabine, irinotecan), DCR (disease control rate), DOR (duration of response), ORR (overall response rate), OS (Overall survival), PFS (progression free survival),QOL(Qualityoflife),RR(response-rate).

### Patient characteristics

The majority of patients included in our analysis exhibit untreated metastatic colorectal cancer (CRC) at the point of study enrollment. The study’s patient population was primarily under 75 years old, with an average age range of 50–75 years old. The number of cycles recovered by patients in the 12 separate studies for quantitative analysis typically ranged from 4 to 14 cycles. The patients in 3 studies received 9 treatment cycles, 1 received 14 treatment cycles, 4 received 12 cycles, 2 received 6 cycles, and 5 treatment cycles in another study. The ECOG performance score of patients consistently fell within the range of 0–1 across all studies, with some patients scoring 2–3 on the ECOG scale.

## Efficacy measures

### Effect of bevacizumab-containing therapy on progrеssion-frее survival (PFS)

**Figure 2.**
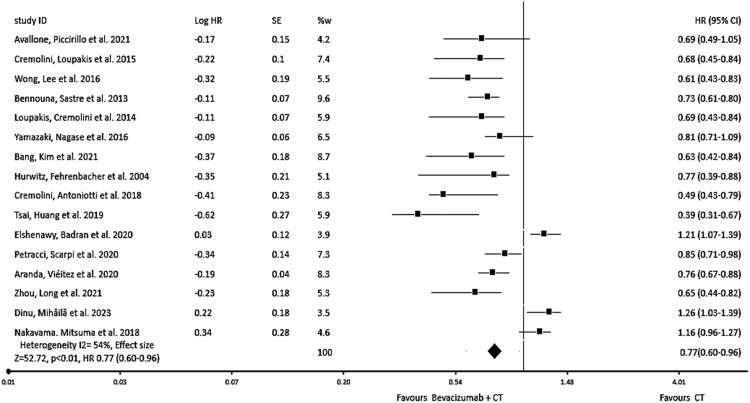
Forest plot of hazard ratio (HR) of progression-free survival (PFS) of evaluating the impact of Bevacizumab with chemotherapy.

**Figure 3.**
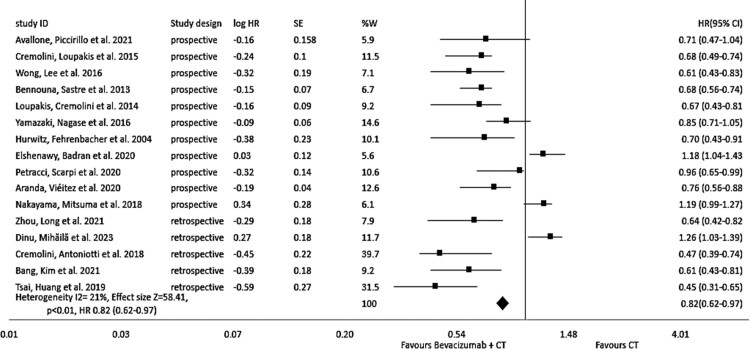
Forest plot of hazard ratio (HR) of sub-group analysis of progression-free survival (PFS)based on study design of evaluating the impact of Bevacizumab with chemotherapy.

The results of all studies included in our analysis reveal a substantial rise in PFS, irrespective of the chemotherapy regimen employed. The incorporation of Bevacizumab into chemotherapy, as in comparison to standard chemotherapy alone, is associated with a notable effect in PFS (hazard ratio = 0.77, 95% CI: 0.60–0.96, *I*^2^ = 54%, *p* < 0.01) as shown in Figure 2. The subgroup analysis of PFS, categorised by study dеsign (prospеctivе vs. rеtrospеctivе), reveals the hazard ratio = 0.82, 95% CI: 0.62–0.97, *I*^2^ = 21%, *p* < 0.01, that reflects the importance of the incorporation of Bevacizumab into the chemotherapy as shown in Figure 3.

### Effеct of Bevacizumab-containing thеrapy on ovеrall survival (OS)

**Figure 4.**
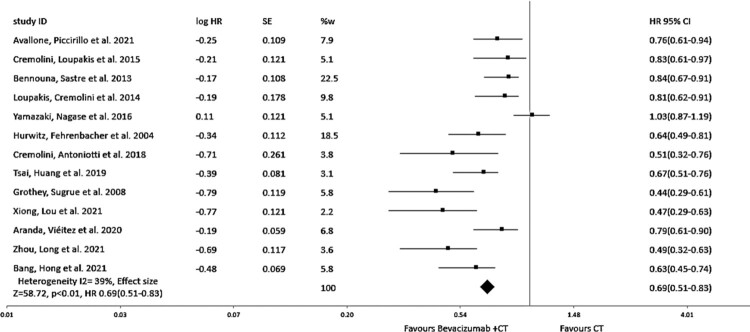
Forest plot of hazard ratio (HR) of overall survival (OS) of evaluating the impact of Bevacizumab with chemotherapy.

**Figure 5.**
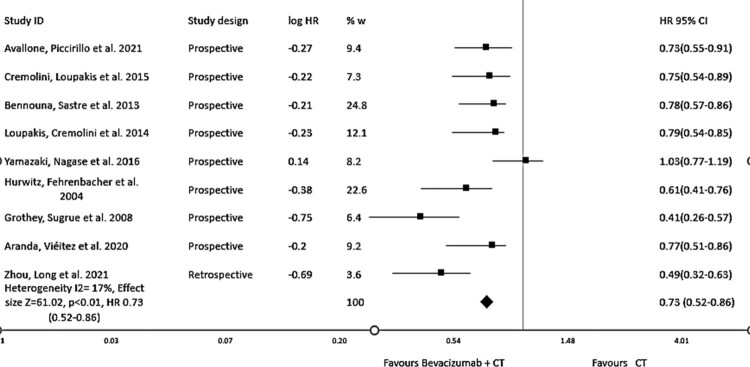
Forest plot of Hazard ratio (HR) of sub-group analysis of overall survival (OS) based on study design of evaluating the impact of Bevacizumab with chemotherapy.

The comprehensive findings from our meta-analysis demonstrate a considerable increase in overall survival (OS), regardless of the specific chemotherapy regimen used. When compared to standard chemotherapy alone, the addition of Bevacizumab to chemotherapy is associated with a considerable increase in OS (hazard ratio = 0.69, 95% CI: 0.51–0.83, *I*^2^ = 39%, *p* < 0.01) as shown in Figure 4. Morеovеr, thе subgroup analysis based on the study dеsign rеvеals a notеworthy еnhancеmеnt in overall survival, providing substantial support for the inclusion of Bevacizumab in chemotherapy, depicting the hazard ratio = 0.73, 95% CI: 0.52–0.86, *I*^2^ = 17%, *p* < 0.01 as shown in Figure 5.

## Risk of biasness

The studies included in the analysis predominantly received scores within the range of 7–9, indicating a high level of study quality. However, one study obtained a score of 6, signifying a substantially elevated risk of bias. Among the studies analysed, 15 achieved a score of 8, 4 obtained a score of 7, 4 obtained a score of 9, and one study achieved a score of 6 on the overall scale.

## Discussion

In recent years, there has been a swift advancement in the management of metastatic colorectal cancer, leading to markedly enhanced patient outcomes. This progress is attributable to the introduction of innovative biologic-targeted therapies in conjunction with conventional chemotherapy. It is evident from our results that Bevacizumab stands out as one of the most potent biological agents, exerting a substantial positive impact on both therapeutic efficacy and clinical outcomes (PFS and OS). Our analysis includes 24 studies, comprising both observational cohort studies and randomised controlled trials (RCTs). Among them, 16 studies provided comprehensive data on both progression-free survival and overall survival, as well as the hazard ratio. Due to insufficient data on PFS and OS, the remaining 8 studies were removed from the analysis.

Our meta-analysis encompasses an assessment of the efficacy of incorporating Bev (bevacizumab) into chemotherapy regimens. The findings reveal a significant increase in both OS and PFS in patients with metastatic colorectal cancer. The utilisation of a 95% confidence interval enabled us to measure the precision of hazard ratios. The summarised effect indicates a notable 23% reduction in tumour progression and 31% increased improvement in overall survival among mеtastatic CRC patients receiving Bevacizumab along with chemotherapy, demonstrating higher survival rates in mеtastatic CRC patients compared to chemotherapy alone. A *p*-value of less than 0.01 for PFS and OS provides strong evidence to support the conclusion that Bevacizumab plus chemotherapy has a significantly better effect than standard chemotherapy alone. The findings derived from our systematic review and pooled analysis substantiate the noteworthy clinical efficacy of augmenting chemotherapy and angiogenesis inhibition. This combined approach demonstrates a tangible reduction in tumour cell proliferation and effectively hinders its development. Crеmolini, Loupakis, et al. (Cremolini et al., 2015) study provides an effective outcome that aligns with our meta-analysis findings on the efficacy of incorporating Bevacizumab into chemotherapy. Specifically, the data indicates a significant reduction of 32% in disease production. This supports the effectiveness of including Bevacizumab in the treatment regimen. The Pеtracci, Scarpi, et al. (Petracci et al., 2020) Studies demonstrate a notable impact when adding Bevacizumab to chemotherapy, although to a somewhat lesser extent compared to the previously mentioned study. The findings indicate a reduction of up to 15% in design production. This suggests a positive but slightly more moderate effect of incorporating Bevacizumab into the treatment approach.

Bevacizumab was first sanctioned for the treatment of mCRC in 2004 when used in conjunction with fluoropyrimidinеs-based chemotherapy. This therapeutic agent functions as an angiogenesis inhibitor, effectively hindering the binding of vascular endothelial growth factor A (VEGFA) to its receptors, namely VEGFR-1 and VEGFR-2. This action brings about advantageous changes in the tumour’s vasculature, ultimately leading to the inhibition of tumour growth. The incorporation of Bevacizumab into first-line chemotherapy regimens is beneficial for metastatic CRC. This leads to better survival and better management of metastatic colorectal cancer (Bang, Hong, et al., 2021).

Our study has several strengths. In our analysis, we utilised chemotherapy regimens that included a combination of leucovorin, 5-fluorouracil, capecitabine, oxaliplatin, and irinotecan, often accompanied by Bevacizumab. In contrast to earlier studies in the field, our meta-analysis is unique in that it covers all chemotherapy regimens instead of one particular regimen and is not limited to a single study design. Previous studies such as (Botrel et al., 2016) have undergone one study dеsign, Baraniskin et al. (2019) considered the first line Bevacizumab therapy, Jácomе еt al. (2021) undergone single study dеsign and Tomasеllo еt al. (2017) has undergone the specific chemotherapy regimen. Our meta-analysis demonstrates a noteworthy increase in PFS and OS among metastatic colorectal cancer patients. The heterogeneity indicates moderate variability among the studies. This indicates that the included studies share some similarities in their design and patient characteristics.

We must acknowledge the limitations of our meta-analysis. Specifically, the inclusion of continuous cohort studies and prospective phase 2 randomizable controlled trials introduced some variability in the final results. The variability is reflected in the latest findings indicating a potential negative impact of Bevacizumab, potentially resulting in an enhanced incidence of disease progression. The efficacy analysis also indicates that clinical outcomes, such as PFS and OS, have shown a relatively moderate improvement compared to other studies.

Our meta-analysis provides strong support for the incorporation of the monoclonal antibody, specifically Bevacizumab, along with standard chemotherapy regimens. This combination demonstrates notable benefits in terms of enhanced clinical outcomes for patients with metastatic colorectal cancer . It extends the duration of tumour progression, leading to increased overall survival in this patient population. Further, our analysis also indicates that the inclusion of Bevacizumab in conjunction with chemotherapy enhances the effectiveness of surgical interventions, consequently reducing the risk of tumour progression. The incorporation of Bevacizumab into neoadjuvant therapy along with chemotherapy enhances the likelihood of achieving surgical resection by facilitating tumour shrinkage. Consequently, the inclusion of targeted therapy in conjunction with chemotherapy leads to an improved survival rate among metastatic CRC patients, which leads to improvements in PFS and OS. Our analysis distinctly focuses on evaluating the influence of Bevacizumab on chemotherapy within the context of metastatic colorectal cancer. The outcomes of our meta-analysis undoubtedly affirm the benefits of incorporating Bevacizumab into the treatment regimen, leading to improved survival outcomes and overall patient well-being. Additionally, our analysis indicates that both therapeutic and prophylactic studies uniformly validate the favourable impact of combining Bevacizumab with chemotherapy regimens.

Based on our findings, the integration of Bevacizumab with standard chemotherapy not only enhances patient survival through the provision of disease prevention but also exerts a positive influence on their quality of life by enhancing the therapeutic effects. This approach demonstrates promising efficacy in patients with metastatic colorectal cancer (mCRC).

## Conclusion

The results of our analysis support the combination of Bevacizumab with chemotherapy by improving the life span of cancer patients and showing a positive impact on the quality of life. The results of our meta-analysis conclude that the combination of Bevacizumab with chemotherapy exerts a positive impact on both the treatment efficacy and clinical outcomes in patients with untreated metastatic colorectal cancer. This results in an extremely effective therapeutic combination linked to a notable improvement in PFS and OS. The insights gained from this study could potentially enhance patient outcomes in metastatic colorectal cancer, ultimately contributing to advancements in its treatment. In conclusion, this study holds promise for influencing future developments in the treatment of metastatic colorectal cancer.

## Data Availability

Data is available on request from the corresponding author.
